# Use of low-dose Aripiprazole to lower the antipsychotic medication - induced hyperprolactinemia.

**DOI:** 10.1192/j.eurpsy.2024.1292

**Published:** 2024-08-27

**Authors:** P. Argitis, A. Karampas, M. Peyioti, T. Koukouras, M. Demetriou, S. Karavia, Z. Chaviaras

**Affiliations:** ^1^Psychiatric, General Hospital of Corfu, Corfu; ^2^Psychiatric, General Hospital of Ioannina, Ioannina, Greece

## Abstract

**Introduction:**

Hyperprolactinemia (HPL) is a condition associated with disturbing consequences. Antipsychotic medications are one of the main causes of nontumoral hyperprolactinemia. Prolactin release in the hypothalamic tuberoinfundibular tract is increased through dopaminergic inhibition, which occurs more frequently with high- potency typical antipsychotics (40%–90%). Less commonly than typical antipsychotics, atypical antipsychotics can also result in hyperprolactinemia. In the presence of symptoms, clinicians frequently struggle with the decision of whether to stop using the suspected offending agent, lower the dosage, switch to another medication, or even add a full or partial dopamine agonist to the patient's current treatment. The issue is exacerbated by the fact that finding a suitable agent for each patient is sometimes a challenging task.

**Objectives:**

Due to the partial D2 receptor agonistic activity of aripiprazole, there is enough dopaminergic tone to continue the inhibition of prolactin release. Aripiprazole has been recommended in literature either as an adjunctive treatment in low doses or as a switch in therapy.

**Methods:**

In the Psychiatric clinic of the General Hospital of Corfu, a low-dose (5mg/day) of aripiprazole is being used as adjunctive therapy in patients with antipsychotic-induced hyperprolactinemia. More specifically in total 42 subjects, 19 male and 22 female, with a mean prolactin level of 862ng/ml, were introduced to the prior therapy. We whereupon conducted prolactin measurements to evaluate the response at the first, the third, and the sixth month of treatment.

**Results:**

Of the 42 subjects, 38 responded with an average reduction of prolactin to the level of 530ng/ml (mean reduction 38,5%).

**Conclusions:**

Having noticed the beneficial effect of low-dose Aripiprazole in patients with antipsychotic-induced hyperprolactinemia, we consider it appropriate that the literature recommendations concerning this additional use of aripiprazole should not be overlooked in clinical practice.

**Disclosure of Interest:**

None Declared

